# Improving Knowledge About Pregnancy for Deaf South African Women of Reproductive Age Through a Text Messaging–Based Information Campaign: Mixed Methods Study

**DOI:** 10.2196/40561

**Published:** 2023-05-22

**Authors:** Hanne Jensen Haricharan, Damian Hacking, Yan Kwan Lau, Marion Heap

**Affiliations:** 1 School of Public Health University of Cape Town Cape Town South Africa

**Keywords:** SMS text messages, cell phones, mobile health, mHealth, health information, health literacy, healthy behavior, maternal health, antenatal care, Deaf, South Africa

## Abstract

**Background:**

Signing Deaf South Africans have limited access to health information and, consequently, limited knowledge about health. Maternal and neonatal mortality rates are high. Cell phone use is high, making it a potentially effective way of communicating about maternal and child health.

**Objective:**

The primary aim of this study was to assess whether an SMS text messaging–based health information campaign could improve knowledge about pregnancy, antenatal care, and healthy living during pregnancy for signing Deaf South African women of reproductive age. The secondary aim was to evaluate the acceptability of such an intervention.

**Methods:**

This study was designed as a pretest-posttest study. A baseline questionnaire assessed participants’ knowledge about pregnancy, antenatal care, and healthy living during pregnancy before an SMS text messaging–based information campaign was conducted. After the campaign, an exit questionnaire was administered containing the same questions as the baseline questionnaire with additional questions on general acceptability and communication preferences. The results were compared between baseline and exit using the McNemar and Wilcoxon signed rank tests. A focus group aimed to obtain further information on the impact and acceptability of SMS text messages. The focus group was analyzed inductively.

**Results:**

The study showed a statistically significant improvement in overall health knowledge among participants. Despite this, some participants found the medical terminology challenging to understand. Several ways of improving SMS text messaging campaigns for the Deaf were identified, including using Multimedia Messaging Services with a person signing messages and linking information campaigns to a communication service that would enable Deaf people to pose questions. The focus group also suggested that SMS text messages might play a role in motivating healthy behaviors during pregnancy.

**Conclusions:**

The SMS text messaging campaign effectively improved Deaf women’s knowledge about pregnancy, antenatal care, and healthy living during pregnancy and has the potential to affect health behavior. This contrasts with a similar study on hearing pregnant women. This suggests that SMS text messages may be particularly effective in improving Deaf people’s health knowledge. However, attention should be paid to Deaf participants’ specific needs and communication preferences to optimize impact. The potential of using SMS text messaging campaigns to affect behavior should be studied.

**Trial Registration:**

Pan-African Clinical Trials Registry (PACTR) PACTR201512001352180; https://tinyurl.com/3rxvsrbe

## Introduction

### Background

This study aimed to assess whether an SMS text messaging–based health information campaign can improve knowledge about pregnancy, antenatal care, and healthy living during pregnancy for signing Deaf South African women of reproductive age. In addition, this study aimed to assess the acceptability of such an intervention. We use Deaf (capitalized) to refer to permanently, sensorily disabled people with congenital or early onset deafness whose first language is sign language (in this context, South African Sign Language [SASL]).

Both maternal and child health are considerable challenges in South Africa, as in many low-and middle-income countries. Since 1994, South Africa has provided free antenatal care for all pregnant women [1]. Maternal deaths have decreased since 2011 but remain high. South Africa’s aim to reduce maternal deaths to 38 per 100,000 births as part of the Millennium Development Goals did not materialize. The latest figure on maternal deaths from 2014 showed 141 deaths per 100,000 births [2].

South Africa also has high rates of perinatal deaths (both stillbirths and early neonatal deaths), with 11 deaths per 1000 live births [3]. The rate increased between 1997 and 2009, after which there was a slight decrease. Since 2010, it has fluctuated relatively highly with no consistent patterns [3]. The leading cause of death has been determined to be maternal factors and pregnancy complications, labor, and delivery, accounting for 21% of deaths [3]. Estimates suggest that 32% and 54% of all maternal and neonatal deaths, respectively, are preventable [4]. Proper health care is critical for preventing these deaths [4].

Regular antenatal care is important for the health of both mother and child as it can identify pregnancy complications [5]. For instance, there is a known association between few antenatal care visits and subsequent preterm birth [5]. According to a report from 2011, a total of 23.4% of assessable maternal deaths among South African women are associated with insufficient antenatal care [2]. Many South African pregnant women access antenatal care late, have low attendance, and experience barriers to receiving quality care [6-9]. This results in challenges in addressing complications during pregnancy. Known reasons for barriers to using antenatal care include the affordability, availability, and acceptability of health care [7-10]. Studies suggest that low use of antenatal care is linked to how pregnant women understand and perceive the benefits of antenatal care to mother and child health [9,10]. In addition, South Africa is reported to have the highest worldwide incidence of fetal alcohol syndrome (FAS) and partial fetal alcohol spectrum disorder [11] because of high levels of alcohol consumption during pregnancy.

Deaf women face challenges in accessing quality health care, and as a result, health outcomes are often worse than for hearing women. There is some evidence suggesting that this is also the case for antenatal care. A study from the United States showed that women with hearing loss have more preterm deliveries and more frequently give birth to babies with low birth weight [12]. Similarly, a study with women with hearing and visual impairments in the United Kingdom demonstrated that disabled women were more likely to have preterm babies [13]. Another US study found no difference in pregnancy outcomes between hearing and Deaf women [14]. A study conducted in Cape Town, South Africa, showed that most Deaf women (96%) accessed antenatal care, though only approximately half initiated care in the first trimester as recommended by the World Health Organization (WHO) [15]. This pattern is consistent with South African hearing women’s antenatal care patterns [9,16-20]. However, 18% of the women taking part in the study with Deaf South African women only accessed antenatal care in the third trimester, citing, among other things, a lack of awareness of the need for antenatal care as a reason for their late booking [15]. Furthermore, the study found that 31% of Deaf women had experienced a miscarriage, compared with 16% of hearing women. We did not find any information on neonatal deaths or FAS prevalence in Deaf South Africans, the target population for this study. However, there is no reason to believe that it should be lower than that of the general population.

Mobile health (mHealth) is the application of mobile technology to address health care issues. The WHO defines telemedicine as healing from a distance using information and communications technology to overcome geographical boundaries and increase health outcomes. mHealth is a subset of telemedicine [16]. In 2011, the WHO defined mHealth as “medical and public health practice supported by mobile devices, such as mobile phones, patient monitoring devices, personal digital assistants (PDAs), and other wireless devices” [17].

In recent years, mHealth has become widespread in high-income and low- and middle-income countries [21-30]. A particular form of mHealth is SMS text messaging.

mHealth has been used to address several health issues. These include distributing health information, affecting behavior change, promoting healthy living, promoting adherence, and reminding patients of appointments [21-30]. However, there is limited evidence on the effectiveness of mHealth and a paucity of knowledge of what it is effective for. In a systematic review, Vodopivec-Jamsek et al [29] concluded that there was limited evidence that health promotion via mobile phone messages effectively supported preventative health care and improved healthy behavior. In contrast, the authors of another systematic review concluded that mobile phones were a promising tool for disease control interventions in low- and middle-income countries [30]. The 2016 review paper by Zhao et al [31], which concerns health apps rather than just SMS text messages, suggests that information conveyed via mobile technology can affect health behavior. Notably, the study only comprised papers from high-income countries and may not necessarily apply to low- and middle-income countries. Nevertheless, the study by Zhao et al [31] is important in noting some of the features that made some health apps effective: they were less time-consuming and perceived to have a user-friendly design.

mHealth has also been used in studies related to maternal and child health [5,32-35]. A systematic review including 15 articles on mHealth initiatives for maternal, newborn, and child health in low- and middle-income countries found that many studies were of poor quality [34]. Of the 15 articles in the review, only 1 study demonstrated improvement in morbidity and mortality [34]. Similar results were found in a systematic review [32]. However, although this review did not observe improvements in maternal and neonatal health outcomes, it noted that some studies improved antenatal and postnatal care attendance. There was also an increase in the number of women who gave birth at a health facility or with a skilled birth attendant. The review also assessed how mHealth interventions affected health knowledge, but the findings on this were inconclusive. Finally, the study stressed some contextual features of campaigns linked to their success. These included using lay terms, communicating in local languages, and considering how to communicate with illiterate populations. The article stressed the latter’s importance as mHealth studies risk increasing health inequities because access to mHealth interventions may be biased toward people of higher socioeconomic status and those residing in urban areas. Another systematic review [33] concluded, contrary to the other papers [32,34], that mHealth for prenatal and newborn health services demonstrated positive outcomes. However, in most cases, the interventions consisted of providing people in rural areas with mobile phones and data to enable them to contact health services. Only 1 study in this review focused on the use of mHealth for health promotion. A review article from 2019 concluded that maternal health was one of the areas in which mHealth was most frequently and successfully used [35].

An important paper to mention is that by Lund et al [5], which reports on a cluster randomized controlled trial in Zanzibar focusing on an SMS text messaging campaign with the primary outcome measure being the number of women who attended 4 antenatal visits as recommended by the WHO. The study found that more women in the intervention arm attended the recommended antenatal visits. Most participants also stated that the educational SMS text messages were helpful during their pregnancies. Furthermore, the authors concluded that the study showed that mobile phones could improve the quality of care by creating awareness about services on the demand side. Similarly, Maslowsky et al [36] found positive evidence for using a mobile phone–based education program in Ecuador. The study noted improvements in breastfeeding, contraceptive use, and infant health but not in postpartum maternal health. A study with second-language English speakers in Cape Town (2012-2013) evaluated an SMS text messaging–based health information campaign on pregnancy and antenatal care [27]. The results showed no statistically significant improvements in health knowledge at exit. However, participants reported that their behavior changed because of the SMS text messaging campaign.

Between 500,000 and 1.5 million South Africans are estimated to be Deaf and use SASL [37]. Communication is a serious barrier to Deaf people’s access to health care and health information with poorer health status [38-43]. Communication problems between Deaf people and health care providers result in delays in diagnosis, missed appointments, repeat visits, misdiagnoses, and misunderstandings [38-42].

Furthermore, Deaf people are often excluded from health research and have limited access to health education programs. Consequently, according to American and Australian studies, their health literacy is poorer than that of the general population [43-45]. Illustrative of this is an American study, which concluded that the Deaf individuals were almost 7 times more likely to have inadequate knowledge about health than hearing participants [45].

There is a paucity of research on health literacy among the Deaf population in low- and middle-income countries. We did not find any studies focusing on health literacy in low- and middle-income countries, including South Africa. However, Deaf South Africans generally have low educational levels, with the average reading level for those attending schools for the Deaf population being lower than the fourth grade [46]. This indicates that their health literacy levels would be equally low.

Health information in South Africa is provided in several ways, including television programs; written material such as pamphlets and posters, which are often displayed at public health clinics; and talks at public health clinics. None of these are easily accessible to Deaf people. The text-heavy written information is inaccessible because of their low literacy and the language level used [46]. Television programs are inaccessible because of the Deaf individuals’ disability, as are talks at the clinics as they are conducted without sign language interpreters. Health communication in sign language has its own challenges. Sign language is a unique language of so-called limited diffusion [47]. This means that it has a limited vocabulary in areas such as health. In other words, certain health concepts may not have an equivalent sign.

Cell phones can be considered an alternative way of communicating health information with signing Deaf people. Most South Africans (75%) have access to a cell phone [48]. Figures are not known for the Deaf population, but we know from our long-term association with Deaf South Africans that SMS text messages have become an important method of communication. For instance, we have previously used SMS text messages to advertise an interpreter service in health care for Deaf people [39].

Limited studies have focused on evaluating SMS text messaging campaigns for the Deaf population. The authors of this paper were involved in a similar study focusing on hypertension knowledge [49], which demonstrated a statistically significant improvement in health knowledge after the SMS text messaging intervention and, thus, indicated that SMS text messages can improve signing Deaf people’s health knowledge. Apart from this study, we did not find any study that evaluated SMS text messaging campaigns with Deaf people. Other health information campaigns focusing on the Deaf population that were identified used videos to convey information on various forms of cancer [50-53]. These studies were conducted in the United States and concluded that videos successfully improved short-term health knowledge.

### Research Justification

This study is based on our knowledge that Deaf people have limited health literacy, but we know from our day-to-day experiences with the Deaf population that they are familiar with using SMS text messages for health care. On the basis of this, we hypothesized that providing Deaf women of reproductive age with easily understandable information about pregnancy, antenatal care, and healthy living during pregnancy via SMS text messages could improve their knowledge. We assumed, drawing on the Health Belief Model [53] and the Theory of Planned Behavior [54], that adequate knowledge is a prerequisite to health-seeking behavior. Furthermore, General Comment 14 on the Covenant on Economic, Social, and Cultural Rights [55], an expert interpretation of the right to health, considers informational access an important component of the right to health. South Africa ratified the covenant in 2015. In line with a human rights framework and behavior theories, we viewed informational access as both a human right and a prerequisite for improving access.

## Methods

We aimed to explore whether an SMS text messaging–based health information campaign for Deaf women of reproductive age could improve their knowledge of pregnancy, antenatal care, and healthy living during pregnancy (Multimedia Appendix 1). The secondary aim was to assess the acceptability of an SMS text messaging campaign among the target population.

### Study Population

The study population was adult Deaf women of reproductive age (18-45 years) whose first language was SASL. Deaf South Africans are a hard-to-reach population. There is no database of this population, and they are geographically dispersed. They also do not use the same health services, meaning that sampling is challenging. Consequently, we used convenience sampling, relying on our contacts with Deaf people and Deaf organizations. Although a larger sample size would have been preferable, preliminary work suggested that we would not be able to include >50 participants in the study.

### Study Design

The study was a mixed methods study consisting of a quantitative component in the form of questionnaires before and after the SMS text messaging intervention. The quantitative part was conducted between June 2013 and November 2013. Although a controlled trial would have resulted in more robust evidence, we believed that such a study design was unreliable as Deaf people in Cape Town form a very close community. This increased the risk of participants sharing information, rendering a controlled trial study design invalid. The second research component was qualitative, a focus group, which explored the results of the quantitative data. The focus group was conducted in May 2014.

The SMS text messaging campaign drew on a campaign with hearing pregnant women, where topics for the campaign had been identified in collaboration with a midwife obstetric unit in a resource-poor setting. A health promotion specialist and an obstetrician also commented on the SMS text messaging campaign. The campaign was amended to address women who were not necessarily pregnant during the campaign and to take Deaf people’s limited literacy and health knowledge into account by simplifying the language. Deaf research assistants vetted the baseline and exit questionnaires (Multimedia Appendices 2 and 3). The campaign was also piloted with Deaf research assistants. The campaign contained 66 SMS text messages distributed over 22 weeks.

### Recruitment and Consent

Recruiting was challenging as the group was geographically dispersed and not registered in a database. We relied on our association with Deaf people and their organizations, primarily the Deaf Community of Cape Town (DCCT), to invite potential participants to information meetings. These meetings were conducted in SASL via an interpreter. In addition, we showed a DVD that explained the research and the consent process in SASL. Potential participants were invited to enroll in the research project on a separate occasion, where Deaf research assistants and translators proficient in SASL answered questions and ensured that participants were informed and consented voluntarily.

Written project information sheets, consent forms, the SMS text messages, and the baseline and exit questionnaires were translated from English into Afrikaans and isiXhosa, the 2 languages used in addition to English in Cape Town. Back translations, in which the Afrikaans and isiXhosa translations were translated into English, ensured consistent information and meaning across the 3 languages.

### Data Collection

After obtaining consent, research assistants administered the baseline questionnaire in SASL. The baseline questionnaire contained 9 questions measuring participants’ knowledge about pregnancy, antenatal care, and healthy living during pregnancy. In total, 4 questions had simple binary answers, such as “Is it important for a pregnant woman to attend pregnancy clinic?” A total of 5 questions had multiple answers, such as “How can a pregnant woman stay healthy during pregnancy?” These questions were presented as multiple-choice questions. In addition to the questions on health knowledge, the exit questionnaire contained questions about communication preferences, acceptability of the SMS text messages, and the experience of receiving the SMS text messages.

A focus group with participants who had received most of the SMS text messages took place 6 months after the exit questionnaire and after analyzing the quantitative data. On the basis of the quantitative results, an interview guide was developed to explore the impact, acceptability, and experience of the SMS text messages. The main author conducted the focus group with the assistance of sign language interpreters and Deaf research assistants. The focus group (the interpreter’s voice-over) was recorded and transcribed.

For the focus group, we invited only participants who had received >80% of our SMS text messages. The research team monitored the delivery of SMS text messages and was aware that not all the SMS text messages were delivered. First, some participants opted out of the campaign or lost their phones. Second, some SMS text messages were not delivered as the phone numbers changed or the phones were left uncharged or switched off for some time. In addition, we found out that it is quite common to share phones among friends and family or change numbers frequently. The loss of cell phones, frequent number changes, uncharged or switched-off phones, and the sharing of phones present confounding factors. As we only became aware of these factors after the quantitative data had been collected, we were not able to control for them.

### Data Analysis

To determine whether there was a change in knowledge, each question and an individual’s total score were analyzed. Each question generated either categorical or continuous data. To determine if the intervention resulted in significant knowledge change, the categorical data were coded into binary outcomes, with “1” signifying correct answers and “0” signifying incorrect answers. The proportion of correct answers was then compared between baseline and exit using the McNemar test for continuous data, and the score for each question was tallied. Correct answers were worth “1,” and incorrect answers were worth “−1.” “Don’t know” was worth “0.” Owing to the relatively small sample size, a Wilcoxon signed rank test was used for all continuous data. The overall score was calculated by tallying the scores of each question at both baseline and exit. The main author analyzed the qualitative data using inductive thematic analysis and discussed it with the other authors.

### Ethics Approval and Informed Consent

This study adhered to the Declaration of Helsinki and was approved by the University of Cape Town Health Science Faculty Human Research Ethics Committee (044/2011). Participants provided written informed consent.

## Results

### Quantitative Results

The study recruited 50 participants at baseline, of whom 8 (16%) were lost to follow-up, leaving 42 (84%) to complete the exit questionnaire. Furthermore, 19% (8/42) of the participants were excluded before the analysis of the survey data as they stated that they did not receive or were uncertain about receiving the SMS text messages. Therefore, the exit survey involved 34 participants.

The mean age of the participants was 34.88 (SD 18) years. Their marital status varied, with 26% (9/34) being married, 18% (6/34) living with a partner, 24% (8/34) living with family, and the rest being single or living with others (11/34, 32%). Most (31/34, 91%) had not finished their high school education. A total of 65% (22/34) were employed but earning a relatively low salary. Most (32/34, 94%) stated that they had been pregnant at least once.

A summary of the demographic profile of the participants is provided in Table 1.

The survey results showed a statistically significant improvement in overall knowledge between baseline and exit (*P*=.002). Tables 2 and 3 show the knowledge improvements for questions with 1 correct answer and multiple correct answers, respectively.

When analyzed individually, 33% (3/9) of the continuous questions showed a statistically significant increase. These were (1) How can a pregnant woman stay healthy during pregnancy? (2) How do drugs and alcohol affect the baby growing in the womb? (3) Should a pregnant woman seek medical help outside her appointments at a pregnancy clinic?

Table 4 shows the improvements for the questions that showed a statistically significant improvement as well as the overall results when all questions were tallied.

The exit questionnaire also explored the acceptability of the SMS text messaging campaign and aimed to assess its usefulness. On a question to determine its usefulness, 94% (32/34) said that the campaign was useful. In line with the survey results, most participants (31/34, 91%) indicated that the SMS text messages had improved their knowledge. A total of 82% (28/34) were unsure, whereas 3% (1/34) of the participants said that it did not improve their knowledge. In total, 65% (22/34) reported that they found the SMS text messages easy to understand, whereas 8% (3/34) did not. Approximately 27% (9/34) were uncertain of how accessible the information was.

One question assessed what participants liked about the SMS text messages. The main quality was that they provided information (31/34, 92%), whereas 76% (26/34) indicated that they considered them trustworthy. A similar number stated that the SMS text messages made them feel cared for. However, 14% (5/34) indicated that the information was irrelevant.

Notably, 59% (20/34) of the participants indicated that they preferred health communication from Deaf organizations, mainly the DCCT. This was followed by information from friends, family, and colleagues, which 19% (6/34) preferred. Written material was preferred by 14% (5/34) of the participants. Interestingly, only 14% (5/34 of respondents indicated that they preferred SMS text messages. Similarly, friends, family, and colleagues were the most common source of information (15/34, 43%). Written material was used by 35% (12/34) of participants, and SMS text messages were used by 32% (11/34). For an overview of communication preferences and acceptability, see Table 5.

**Table 1:** Demographics for participants in the exit survey (N=34).

			Values
Age (years), mean (SD)			35 (18)
**Marital status, n (%)**			
	Divorced	0 (0)	
	Married	9 (26)	
	Single, living alone	5 (15)	
	Single, living with family	8 (24)	
	Single, living with a partner	6 (18)	
	Single, living with nonfamily	5 (15)	
	Widow	1 (3)	
**Educational level, n (%)**			
	Below grade 7	15 (44)	
	Between grade 7 and grade 12	16 (47)	
	Passed high school	2 (6)	
	Post–high school education	1 (3)	
**Employment status, n (%)**			
	Employed	22 (65)	
	Pensioner	1 (3)	
	Unemployed	7 (21)	
	Not looking for employment	1 (3)	
	Never worked	3 (9)	
**Monthly income, n (%)**			
	None	7 (21)	
	Pension or social grant	8 (24)	
	<ZAR 4000 (US $222.59)	14 (41)	
	Between ZAR 4000 and ZAR 10,000 (US $222.59-$556.48)	2 (6)	
	>ZAR 10,000 (US $556.48)	0 (0)	
	No answer	3 (9)	
**Preferred language for reading and writing, n (%)**			
	English	26 (76)	
	Afrikaans	5 (15)	
	isiXhosa	3 (9)	
**Gravidity, n (%)**			
	0 pregnancies	2 (6)	
	1 pregnancy	8 (24)	
	2 pregnancies	11 (32)	
	3 pregnancies	9 (26)	
	4 pregnancies	2 (6)	
	5 pregnancies	2 (6)	

**Table 2:** Knowledge scores for questions with binary answers presented as the number of people with adequate knowledge at baseline and exit (N=34).

Question	Baseline, n (%)	Exit, n (%)	*P* value
Is it important for pregnant women to attend pregnancy clinics?	30 (88)	33 (97)	.25
Should a pregnant woman ask for the results of her pap smear (a test that checks for cancer of the mouth of the womb)?	21 (62)	23 (68)	.80
Why do the nurses test the urine and blood pressure when pregnant women attend a clinic for pregnancy?	13 (38)	14 (41)	>.99
Why should a pregnant woman take folic acid tablets (which she gets at the clinic) during pregnancy?	5 (15)	12 (35)	.12

**Table 3:** Knowledge scores for questions with multiple correct answers (N=34).

Question (number of correct answers/number of options) and number of correct answers selected			Baseline, n (%)		Exit, n (%)		*P* value	
**Why do the nurses test the blood of pregnant women? (3/7)**								.96
	0	19 (56)		22 (65)				
	1	13 (38)		6 (18)				
	2	2 (6)		6 (18)				
	3	0 (0)		0 (0)				
**How can a pregnant woman stay healthy during pregnancy? (4/9)**								.03
	0	8 (24)		5 (15)				
	1	6 (18)		3 (9)				
	2	9 (26)		3 (9)				
	3	6 (18)		11 (32)				
	4	5 (15)		12 (35)				
**How do drugs and alcohol affect the baby growing in the womb? (2/3)**								.048
	0	22 (65)		10 (29)				
	1	9 (26)		24 (71)				
	2	3 (9)		0 (0)				
**Should a pregnant woman seek medical help outside her appointments at the pregnancy clinic? (2/4)**								.001
	0	22 (65)		8 (24)				
	1	10 (29)		15 (44)				
	2	2 (6)		11 (32)				
**What are the signs of labor? (3/6)**								.16
	0	16 (47)		14 (41)				
	1	12 (35)		9 (26)				
	2	5 (15)		7 (21)				
	3	1 (3)		4 (12)				

**Table 4:** Knowledge scores for questions with a statistically significant improvement.

Question	*P* value
How can a woman stay healthy during pregnancy?	.03
How do drugs and alcohol affect the baby growing in the womb?	.048
Should a pregnant woman seek medical help outside her appointments at a pregnancy clinic?	.001
Overall score	.002

**Table 5:** Acceptability of SMS text messages (N=34).

Question	Participants, n (%)
Read all or most of the SMS text messages	34 (100)
Felt that the SMS text messages improved their pregnancy knowledge	28 (82)
Thought that the SMS text messages were easy to understand	31 (91)
Found the SMS text messages useful	31 (91)
Found the SMS text messages trustworthy	28 (82)
Felt that somebody cared about them	25 (74)
Found the SMS text messages entertaining	20 (59)
Found the information in the SMS text messages not helpful	3 (9)
Found the SMS text messages to be irritating	1 (3)
Did not like the SMS text messages	1 (3)
Felt that the SMS text messages were the best way of giving information to Deaf people	5 (15)

### Qualitative Results

A total of 11 women took part in the focus group. This is equivalent to 30% (11/34) of participants in the exit survey. In total, 36% (4/11) of those participating in the focus group indicated that they had been pregnant during the SMS text messaging campaign. The demographic data of the focus group participants were largely similar to those of the participants in the survey.

The focus group confirmed that Deaf people have a health knowledge gap that SMS text messaging campaigns can potentially address. Participants explained that they faced challenges in accessing health information. Consequently, the focus group was used to seek more information and clarity on issues raised during the SMS text messaging campaign.

The focus group also confirmed the survey results by indicating that the SMS text messaging campaign gave participants important new information and raised their awareness. A participant summarized it as follows:

I found all the messages interesting, and I learnt things I never knew.

Participant 1

Another participant compared the SMS text messages with other information sources such as pamphlets handed out at the clinics:

At the clinic, they always have people who come and teach health information, but we never have an interpreter for us. They give us pamphlets with complex language...So, it is very hard for us to understand. So, it was easy for us to learn with the UCT’s (University of Cape Town) SMSs.

Participant 2

In general, participants argued that their understanding was facilitated by the SMS text messages using simple language and being brief, as illustrated by the following quote:

For me, it’s easy to understand UCT’s SMSs because they have simplified the terminology. (It is easier) than having to read a pamphlet from the clinic that is made for hearing people and that is made for academic people. That terminology I really don’t understand as much as I understand the SMSs.

Participant 3

However, some participants argued that they found the SMS text messages challenging to understand, mirroring the survey, where 27% were uncertain of how accessible the information was. Medical terminology was particularly challenging. Words such as *abdominal* and *fetal alcohol syndrome* were noted as being particularly difficult. A participant explained that there was no sign for the word *abdominal*. Referring to an SMS text message about the possible harmful effects of alcohol in increasing the risk of FAS, another woman explained the following:

So, that word, I don’t understand. (The baby) might be born with what? So I think I still need to clarify and learn more about that because I understood from the SMSs that when a woman is pregnant, she must not drink because “this” (foetal alcohol syndrome) could happen to the baby. But I don’t know what could happen to the baby.

Participant 4

Another participant had a different experience with the SMS text message referring to FAS. She explained that she had seen posters at the clinic saying that pregnant women should not drink but had not understood the reason. According to her, it was different with the information in the SMS text messages:

During pregnancy, we go to the hospital, and you will see posters around with a picture of a pregnant woman and then a bottle of alcohol saying, “you must not drink alcohol,” but you won’t know how serious it is. How can it affect the baby? Then you’ll find yourself continuing drinking while you are pregnant, but with UCT’s SMSs it was more clear. We understand that we must not take alcohol because it can affect the baby.

Participant 5

Participants who found the SMS text messages difficult to understand talked about their poor educational background resulting in their difficulties with written information. Several participants suggested that the SMS text messages could be made more accessible by using simpler language. Others argued that the SMS text messages could use “sign language structure,” a form of informal writing that uses the unique structure of sign language, which has its own word order and grammar:

So, if you can just make it like easier for us Deaf people, you can put it in sign language structure. That will actually be helpful for us.

Participant 6

However, there was disagreement on this point, with some participants arguing that the SMS text messages should be in plain English but at the same time introduce medical terminology, which should be explained. They reasoned that it was important to retain medical terminology in the SMS text messages as this could improve their knowledge of it. Knowing medical terms, they argued, would make it easier for them in a health care setting, as illustrated in the following quote:

The SMSs that we received, they helped us a lot because before UCT sent the SMSs, we didn’t know anything. Even when we go to the doctor, we don’t know; we don’t notice when something is about to happen. But then, when we received the SMSs, we could see that this had happened to me before, but I didn’t know. I couldn’t go to the clinic because I didn’t know what was happening. The SMSs helped us a lot to understand and to see what we missed on...so the SMSs were really helpful.

Participant 7

The use of pictures in the SMS text messages was also advocated for by participants, who explained that Deaf people are more familiar with visual communication, which can help them understand written language:

If UCT’s SMSs could be accompanied by pictures. It’s easy when they speak about feet or about the nose; then there will be a picture of the nose.

Participant 8

Another theme raised in the focus group was that the SMS text messages were insufficiently detailed, and participants requested more information. This is not surprising when considering that the SMS text messages were limited to 160 characters. Participants suggested that the SMS text messaging campaign be combined with a communication service where they could write back and ask to have a word explained further or obtain more information.

An important suggestion for improvement was using signed video messages (Multimedia Messaging Services [MMSs]). Participants suggested that this was a better mode of communication for Deaf people than written SMS text messages:

I did not like the fact that it was written. I would have preferred videos of a sign language interpreter. We should have been able to obtain it (the information) via sign language.

Participant 9

A theme that also indicated Deaf people’s preference for visual communication was a preference for the health dramas organized by the DCCT. Participants said that they preferred these dramas as they found them both informative and entertaining. Furthermore, many participants argued that using different communication modes would strengthen the campaign’s effectiveness:

I would say DCCT is good, but it’s more good [sic] when you mix DCCT with the SMSs that you receive from UCT because you will see them having a drama at DCCT and then it will match what you will receive from the SMSs. So, it’s like a learning curve that is big. So together, they make a lot of sense.

Participant 10

Notably, the quotes highlight that the combination of different communication modes can be useful as it bridges the gap between a form of communication familiar to Deaf people—signs and visual communication—with the medical world dominated by words and medical terminology. There was consensus among focus group participants that effectiveness would likely improve by using different methods to convey the same health information. This was related not only to the dramas and SMS text messages but also to the pamphlets they received at the clinics.

Similarly, the repetition of information, built into the SMS text messaging campaign, improved effectiveness. Most participants felt that repetition was beneficial for retaining the knowledge conveyed:

It is good because it is like you receive a reminder. Maybe you will get an SMS first, and then with time, because you receive a different SMS...and then when that SMS comes again, it’s like a constant reminder that you must not do this. So that’s why it is good.

Participant 11

Similarly, many participants said that they kept the SMS text messages on their phones for easy retrieval, and some wrote down the SMS text messages in a notebook. This enabled them to use the messages to gain further information or seek clarity on the meanings that they did not understand. Being able to reread information also seemed to be beneficial, as the following quote indicates:

Some of them were a bit hard for me to understand, but now because they are on my phone, I can always go back and refer. So now the words I didn’t understand a bit, now I can understand because it being there, I can always refer back.

Participant 12

Our primary aim was to improve knowledge among participants, and our study population comprised women of reproductive age. However, 36% (4/11) of the focus group participants had been pregnant during the SMS text messaging campaign. All women who had been pregnant (4/4, 100%) commented that the campaign had affected their health-seeking behavior. This suggests that SMS text messages may also be an effective way of influencing healthy behavior. In particular, their eating habits were altered. A woman said that nurses had complained about her “behaviour” during her first 2 pregnancies but explained that she changed after receiving the SMS text messages:

So UCT helped me to understand that during my pregnancy, I need to eat healthy food. I need to cut the drinks aside, fats aside. So, it helped me a lot, and I was able to have a normal pregnancy until I delivered.

Participant 13

In a similar way, the following woman explained how the SMS text messaging campaign assisted her in having a healthy pregnancy:

Ok, on my first pregnancy, I was going to the hospital alone, and then I didn’t understand what to eat, and then all the time, nurses and midwives would shout at me, “why is your blood pressure so high?,” “Why are you not taking care of yourself?” And then I fell pregnant again. And still, I didn’t understand because UCT was not there, and there were no sign language interpreters. But then, when I went for the third time, luckily, I had the SMSs, and I had interpreters with me. And then I understood what is important to eat and all that. So, I’m grateful to UCT because I managed to change my lifestyle.

Participant 14

None of the women commented that the campaign influenced their clinic attendance. Furthermore, it is important to note that a healthy pregnancy and delivery for the aforementioned woman were also linked to the availability of a sign language interpreter during clinic appointments and delivery.

## Discussion

### Principal Findings

The results showed that participants’ knowledge improved at exit, suggesting that SMS text messages can improve Deaf women’s knowledge about pregnancy, antenatal care, and healthy living. A similar SMS text messaging–based health promotion campaign with hearing pregnant women attending an obstetric unit in a similar resource-poor setting showed no statistically significant improvement in knowledge after the SMS text messaging campaign [27]. Notwithstanding the fact that the women in the hearing study were pregnant, whereas the participants in the Deaf study were women of reproductive age, the difference between the 2 studies could suggest that SMS text messaging campaigns are particularly effective for Deaf populations. A possible explanation for the difference could be that Deaf participants’ baseline knowledge was lower than that of participants in the hearing campaign. Therefore, the potential for impact was greater. Accessing health information is challenging for Deaf people, and methods that address these challenges, such as SMS text messages, may be particularly well suited for this group. This argument is supported by a similar study on hypertension among Deaf South Africans, which also showed an overall statistical improvement between baseline and exit [48]. In contrast, a linked study with hearing patients with hypertension did not show statistically significant improvements. Thus, although 2 SMS text messaging campaigns among hearing women in the same geographical locality did not result in improvements, 2 campaigns among Deaf populations did. We have not identified any other SMS text messaging studies with the Deaf population.

In addition to noting the overall statistically significant improvement, it is worth noting that knowledge improved for all 9 questions, although the gains were statistically significant for only 3. The first individual question that showed a statistically significant improvement was about healthy living during pregnancy, focusing on healthy eating, exercise, smoking, and alcohol consumption. The second individual question that showed statistically significant knowledge improvement related to how drugs and alcohol affect the unborn baby. This is particularly important to take cognizance of given that the Western Cape province, where this study took place, has the world’s highest rate of FAS [11]. The woman who argued that new knowledge let her “to put the drinks aside” exemplifies how knowledge about the effects of alcohol was linked to self-reported behavior change.

Finally, a question assessing knowledge of when pregnant women should seek medical assistance outside clinic appointments registered a statistically significant improvement. The information conveyed in the SMS text messages and assessed in the questionnaire focused on symptoms such as bleeding, persistent frontal headache, sudden swelling, and lack of fetal movements. Given South Africa’s many avoidable maternal deaths [4], it is important to explore ways of addressing these issues, including for Deaf women.

It is also important to note the research that found that 18% of Deaf pregnant women only seek antenatal care in the third trimester [15], partly because they lack knowledge of the importance of antenatal care. Furthermore, they have twice as many miscarriages as hearing South African women, which could be linked to not seeking antenatal care in emergencies. The literature has suggested that SMS text messages successfully got hearing women to attend 4 antenatal visits as recommended by the WHO [5,32], showing that SMS text messages can affect attendance patterns. Other studies have found that low antenatal attendance is linked to how women perceive benefits to mothers and children [2,10]. The fact that women in this study indicated that they understood the importance of accessing emergency care is a testimony to the impact that SMS text messaging campaigns could have on antenatal care attendance patterns. This should be studied further.

Despite the main finding of this study, it is important to note that 27% of participants were unsure of how accessible the information in the SMS text messages was. This is not surprising considering their low health and general literacy and the fact that sign language often lacks health terms [47]. Medical terminology was complicated for some participants, as illustrated by the quote on FAS. This affected the overall success of the campaign. This points to the importance of taking cognizance of Deaf people’s needs and preferences for visual communication, such as pictures and posters, when designing health information campaigns. In particular, the proposal to use signed MMSs to disseminate health information at regular intervals seems promising.

An important advantage of signed MMSs is that medical terms can be better explained and more detailed information can be conveyed. However, signed MMSs may also have disadvantages compared with written SMS text messages. Some participants argued that it was essential for them that the written SMS text messages introduced them to written medical terminology, which they found helpful in clinical settings where they often rely on written notes to communicate with their health care provider. Although sign language interpreters can improve patient-provider communication [38], the reality for most Deaf South African patients is that medical encounters occur without a sign language interpreter, resulting in difficulties in provider-patient communication [39]. In light of this, it is easy to understand why participants in the focus group stressed the importance of improving their understanding of written medical terminology.

Here, it is important to consider that sign language is a language of limited diffusion. It is worth considering initiatives such as the Australian Medical Signbank [47], which addresses this by developing signs related to health and medicine. Similar initiatives for SASL could strengthen health communication between providers and Deaf patients and be useful in signed MMS-based campaigns. However, in the absence of medical signs and sign language interpreters in medical consultations, Deaf South Africans’ reliance on written notes for communicating with their health care providers means that knowing written medical terminology is an advantage. Whether signed MMSs and written SMS text messages are preferable should not be treated as an either-or question. Signed MMSs may be superior in imparting health information, whereas SMS text messages can introduce the Deaf individuals to written medical terminology essential in patient-provider communication. A possible solution could be to combine signed and written messages. Interestingly, a South African health app for the Deaf population is being developed and is considering combining text and signs [56]. However, data price should be considered when using MMSs as data costs are a barrier for Deaf South Africans [56].

Our project has shown that SMS text messaging campaigns are a valuable method to communicate health information to the Deaf population that currently seems underused. Other methods used with this target group include internet training workshops, peer education programs, and videos. The latter has successfully raised cancer awareness [50-53]. Compared with written SMS text messages, videos communicate in sign language and, thus, have similar advantages to MMSs. However, it is important to consider the context in which these video screenings took place. Although video screenings may work in the United States, where the studies were conducted, video screenings with Deaf South Africans could be challenged by limited geographical reach.

Furthermore, video screenings are one-off events, and retaining the information may be difficult. The studies on videos assessed knowledge 2 months after the screenings, and it is impossible to determine long-term knowledge retention, as it was in our study. Interestingly, participants in our studies mentioned that they kept the SMS text messages on their phones or wrote down the content in a notebook to be able to retrieve the information. They also noted that they appreciated that information was repeated as it aided their understanding. In some of the video studies, the video was uploaded to the internet, making it accessible for more than one viewing. However, it may present a barrier in the South African context, where most Deaf people have limited internet access, limited computer literacy skills, or cannot afford to access the internet [56]. In this context, MMSs may be a preferable option.

The secondary aim of this study was to assess the acceptability of the SMS text messaging campaign. The focus group suggested that SMS text messages were an effective and acceptable way of addressing participants’ health knowledge gap. Participants appreciated the informative value of the SMS text messages, their briefness, and the accessible language. This is in line with the study by Zhao et al [31], which argued that design issues are important for successful mHealth campaigns. The features included not being time-consuming and being user-friendly and easily accessible. However, it is also important to acknowledge that 27% of our participants were unsure of how accessible the information was. This points to pilot campaigns’ importance in assessing and addressing language and design issues.

A total of 12% (4/34) of the participants, who were pregnant during the study, reported behavior change. No clinical data were available to confirm this, and it is difficult to know whether the changes were real or reflected social desirability. However, self-reported behavior change should be seen at least as a desire or intent to change behavior. Furthermore, participants linked their new knowledge to behavior change, indicating that SMS text messages could be the first step in health-seeking behavior. Theoretically, this aligns with the Health Belief Model and the Theory of Staged Behavior [53,54,57].

Moreover, these tentative findings are similar to those of a study conducted by the same authors, which evaluated an SMS text messaging campaign on hypertension for the Deaf individuals [49]. This study showed self-reported behavior change among participants with hypertension. Although the results of both studies should be interpreted with caution because of the small sample sizes, they point to behavior change as an important area for future studies.

### Areas for Further Research

Campaigns that combine different communication methods such as dramas, posters, pamphlets, and signed MMSs or written SMS text messages should be explored as encountering information in different ways seemed to aid understanding and knowledge retention. Signed MMSs, either on their own or in combination with written SMS text messages, seem to be a promising avenue for further research. In addition, combining information campaigns with an interactive communication service that enables Deaf participants to raise questions that can provide more detailed information should be considered. Such campaigns could benefit from the development of new signs for health and medical terms.

This study suggests that SMS text messaging campaigns may affect behavior change, but more rigorous studies are needed to investigate this area. In the field of antenatal care, several potential research areas were identified, including health care attendance in emergencies. Campaigns with Deaf women could assess the impact of SMS text messaging and mixed information campaigns on health-seeking behavior. A particular area is using mHealth to create awareness of the harmful effects of alcohol, which could potentially affect FAS incidents.

Longitudinal studies with a larger sample size and, if possible, randomized controlled designs would be useful to determine the potential of SMS text messaging campaigns among Deaf populations, improving knowledge and health behavior. Evidence of their potential effects on behavior would be strengthened by including clinical measures.

### Strengths and Limitations

The main strength of this study is that it demonstrates that SMS text messages can improve health knowledge about pregnancy, antenatal care, and healthy living during pregnancy for Deaf South African women of reproductive age. Along with a similar paper on hypertension knowledge among Deaf South Africans, it strengthens the argument that SMS text messages can address Deaf people’s health knowledge gap. We have not been able to identify other studies that show this. Hence, this study contributes to understanding ways of improving Deaf people’s health knowledge. This study also contributes to understanding how information campaigns for the Deaf population can be improved by paying attention to design aspects.

Loss to follow-up presents a limitation. The relatively small sample size may also limit the generalizability of our findings. Possible selection bias should be noted. Possibly, those who decided to participate had a particular interest in the topic. Similarly, it is conceivable that loss to follow-up resulted from participants finding the SMS text messages challenging to understand or uninteresting. It should also be noted that the study was conducted in a metropolitan area, which may affect the generalizability of our findings. A further limitation is that there may be volunteer bias among those attending the focus group. The possibility exists that only those who appreciated the SMS text messaging campaign decided to accept the invitation to attend the focus group. Behavior change was self-reported, was not supported by clinical data, and could reflect social desirability. The exit questionnaire for this SMS text messaging campaign was completed 3 weeks after the campaign ended, making it difficult to assess how much knowledge would be retained in the long term. Confounding factors such as loss of phones, uncharged phones, and sharing of phones should be controlled for in future studies.

### Conclusions

Deaf people have limited access to health information. This study documents that SMS text messages were an effective and acceptable way of imparting information about pregnancy, antenatal care, and healthy living to Deaf women of reproductive age. SMS text messages for Deaf people need to pay attention to language and medical terminology. SMS text messaging campaigns for the Deaf population can be improved by paying close attention to Deaf people’s communication preferences, for instance, using pictures or signed MMSs. Ideally, SMS text messaging campaigns for the Deaf population should be combined with an interactive form of communication that would enable Deaf participants to seek further information and ask questions. Furthermore, campaigns should explore the combination of different communication forms using signs and written language. Future research should examine whether SMS text messages can motivate behavior change.

## Appendix

Multimedia Appendix 1 SMS text messaging campaign.

Multimedia Appendix 2 Baseline questionnaire.

Multimedia Appendix 3 Exit questionnaire.

## Abbreviations

DCCT: Deaf Community of Cape Town
FAS: fetal alcohol syndrome
mHealth: mobile health
MMS: Multimedia Messaging Service
SASL: South African Sign Language
WHO: World Health Organization

## Data Availability

The data set used during this study is available from the corresponding author upon reasonable request.
