# Supplementary material for: Improving Knowledge About Pregnancy for Deaf South African Women of Reproductive Age Through a Text Messaging–Based Information Campaign: Mixed Methods Study
Source: JMIR Pediatr Parent. 2023 May 22;6:e40561. doi: 10.2196/40561 (PMC10242462; doi:10.2196/40561)
Supplement: Multimedia Appendix 1 [file pediatrics_v6i1e40561_app1.docx]

Multimedia Appendix 1. SMS text messaging campaign.

| Welcome: you have signed up for SMSs about pregnancy. Save this number on your phone. |
| --- |
| Did you know? If you do not want to continue receiving SMSs, please send a "please call me" to the following number: |
| Pregnancy does not mean you are sick, but it is important for pregnant women to take care of their health. |
| Healthy mum: Pregnant women must attend all visits to the clinic and must look after themselves and baby. |
| Healthy baby: During the first 12 weeks of pregnancy, baby’s organs (heart & lungs) will be formed. It is important for pregnant women to stay healthy. |
| Healthy mum: It is important not to smoke, drink alcohol, or use drugs when you are pregnant. This is not good for the baby. |
| Healthy mum: Pregnancy is not a sickness, but you must attend check-ups at the clinic. Medical care is important for you and baby. |
| Healthy mum: During early pregnancy woman may feel sick and vomit. If a pregnant woman has any other problems, she must speak to a nurse. |
| Healthy mum: Heartburn, backache, swollen hands & feet are normal in pregnancy. But a pregnant women should tell the nurse if it carries on too long. |
| Healthy baby: X-ray may harm baby in the womb. If the health staff want to take x-rays, pregnant women must explain that they are pregnant |
| Healthy mum: Pregnant women need to report any unusual symptoms during the pregnancy to the nurse at the clinic. |
| Healthy baby: Clinic gives pregnant women iron & folic acid tablets to ensure baby’s health & development. Pregnant women must take these tablets daily. |
| Healthy baby: Pregnant women need to take iron tablets. Iron ensures that the baby gets enough oxygen, helping the baby grow and develop normally. |
| Healthy baby: Folic acid tablets lowers danger of serious birth defects (body & mental). Pregnant women must take these tablets every day. |
| Healthy mum: It is a good idea for pregnant women to take vitamins during pregnancy. |
| Healthy mum: Pregnant women's health is important for baby. Infection from sex germs (Sexually Transmitted Infection or STI) is harmful for mother & baby. |
| Healthy mum: Pregnant women, who have STI, need treatment. Treatment is important for mother & baby. |
| Healthy mum: Clinic does pap-smears for pregnant women over 30 years or HIV+. Pap-smear checks if the cervix (mouth of the womb) is healthy. |
| Healthy mum: Pap-smears can detect cells that are not normal & may later become cancer of the mouth of the womb - but it can be cured if detected early. |
| Healthy mum: If clinic does a pap-smear, pregnant women need to ask for the results at their next clinic visit. |
| Healthy mum: Sister checks bloods to see if pregnant women have an infection from sex germs (STI), diabetes or HIV and need extra care. |
| Healthy mum: Sister may also check to see if a pregnant woman has enough iron in her blood. This is important to keep her and baby healthy. |
| Healthy mum: Tests are important. Treatment for too little iron in the blood or for infection from sex germs (STI) make sure mum & baby are healthy. |
| Healthy mum: Pregnant women need to ask for the result of their pap-smear. The clinic will not contact them to tell them the result of the pap-smear. |
| Healthy baby: Drinking alcohol while pregnant, (ESPECIALLY first 12 weeks!) causes baby to have problems & be sick. Pregnant women must not drink alcohol! |
| Healthy baby: When pregnant women drink alcohol, baby may get a sickness. Name of the sickness is foetal alcohol syndrome or FAS. |
| Healthy baby: Foetal alcohol syndrome gives children learning difficulties and behavioural problems for life. |
| Healthy baby: Taking drugs while pregnant can cause serious problems for baby especially during first 12 weeks. Pregnant women must not take drugs. |
| Healthy baby: Even after 12 weeks, taking drugs can cause the baby not to be normal. Pregnant women should speak to the nurse if they use drugs. |
| Healthy mum: When a pregnant woman smokes, the baby does not get enough air and it can affect baby’s growth. |
| Healthy mum: Many medicines are dangerous to use during pregnancy. A pregnant woman should tell her doctor, nurse and chemist that she is pregnant. |
| Healthy mum: A pregnant woman should only take medicine that a nurse or doctor has told her to take. |
| Healthy mum: It is important for a pregnant woman to look after herself and ensure that she has support. |
| Healthy mum: Remember if cervical cancer is treated early, it can be cured. It is important to know the result of a pap-smear. |
| Healthy baby: The mother must feel baby kicking 10 times daily. If baby does not kick 10 times daily, mother must go straight to clinic. |
| Healthy mum: Women who have a pap-smear must ask for the results. It detects cancer of the mouth of the womb & mum can get early treatment. |
| Healthy mum: Exercise in pregnancy is important. Keeping fit will help make a pregnant woman more comfortable and have an easier birth. |
| Healthy mum: Exercise is great, but if a pregnant woman is ill or has pregnancy problems she should speak to her doctor before she starts exercising. |
| Healthy mum: Sex during pregnancy is safe, but if a woman has pain or is worried about it, she should discuss it with the nurse or doctor. |
| Healthy mum: Nurse checks urine & blood-pressure every visit to ensure pregnant women don’t get pre-eclampsia (high blood pressure & kidneys not working). |
| Women with high blood pressure are at higher risk of developing pre-eclampsia and will need extra care. They may need to change their medication. |
| Healthy mum: If a pregnant woman starts bleeding or water leaks, she should go to the Maternity Unit. |
| Healthy mum: If a pregnant woman has unexplained pains, she needs to seek medical help/go to the MOU. It is open 24 hours every day. |
| If a pregnant woman has many headaches in the front of her head, sudden swelling of hands, feet & face, she must phone for an ambulance immediately. |
| Healthy mum: Healthy eating habits are important for both mother and baby. A pregnant woman should make sure she gets lots of fruit, vegetables & milk. |
| Healthy mum: A pregnant woman should drink lots of water, milk & fruit juice to stay healthy. She must try to stop coffee & tea, but rooibos tea is fine. |
| Health mum: A pregnant woman who is HIV-positive needs extra care during pregnancy. |
| Healthy mum: The clinic gives medicine to HIV-positive women and their babies to protect baby from HIV. |
| Healthy baby: A pregnant woman who is HIV-positive must choose between breast milk OR formula milk. Mixed feeding puts baby in danger of getting HIV. |
| Healthy baby: During the last 3 months of pregnancy baby gets very big in the womb and gets ready to be born. |
| Healthy mum: It is normal being tired during pregnancy, especially during the last 3 months. Pregnant women should get plenty of rest. |
| If a pregnant woman has many headaches in the front of her head, sudden swelling of hands, feet & face, she must phone for an ambulance immediately. |
| Healthy mum: A month before the baby will be born, the pregnant mother should slow down and prepare for the birth of her baby. |
| Healthy mum. Breastfeeding is better, easier and cheaper. But not all women can breastfeed. |
| Healthy mum: Breast is best for babies. It is healthier and it has soldier cells that help protect babies against illnesses. |
| Healthy mum: Breastfeeding is good for mothers too. It may protect against getting cancer later in life. |
| Healthy mum: Breastfeeding can help women lose the ‘baby fat’ quickly after birth. |
| Healthy mum: If a woman wants to breastfeed, but has to go back to work soon after birth, she should discuss options with the Sister. |
| Healthy baby: A mother can save breast milk for the baby in a cup. In that way, baby will be able to get breast milk, when her mother is not with her. |
| Healthy mum: Pregnant women must go to Maternity Unit/clinic immediately if she has lots of headaches in front of head, sudden swelling of hands, feet & face. |
| Healthy mum: Having support during labour is important. A pregnant woman should make plans to have support from a partner, friend or family member. |
| Healthy mum: Having a new-born baby is exciting and tiring. Pregnant women should organise support when they get home from hospital. |
| Healthy mum: Bleeding, water breaking, or labour pains are signs of labour. This means it is time for the pregnant woman to go to the MOU. |
| Healthy mum: When labour starts, a woman should check time between labour pains. 3 labour pains in 10 minutes means she should go to the MOU. |
| Health tips are ending. You will not receive SMSs anymore. We hope you learned a lot about pregnancy. |
