# Supplementary material for: Improving Knowledge About Pregnancy for Deaf South African Women of Reproductive Age Through a Text Messaging–Based Information Campaign: Mixed Methods Study
Source: JMIR Pediatr Parent. 2023 May 22;6:e40561. doi: 10.2196/40561 (PMC10242462; doi:10.2196/40561)
Supplement: Multimedia Appendix 2 [file pediatrics_v6i1e40561_app2.docx]

**Multimedia Appendix 2. Baseline questionnaire.**

**1. Reference__________________**

**2. Consent signed?**

1. Yes

**4. Is it important for a pregnant woman to attend pregnancy clinic? Choose one answer.**

1. Yes
2. No, not if you are healthy
3. Don’t know

**5. Why do the nurses test the blood of a pregnant woman?** **Choose as many answers as you like.**

1. To make sure that a pregnant woman and her baby have enough red blood cells to keep them healthy
2. To make sure that a pregnant woman her partner do not have a sexually transmitted disease
3. To test for sugar
4. To test for HIV/AIDS
5. To test for cholesterol
6. To test blood type – if negative the baby will need medication after birth
7. Don’t know

**6. Should a pregnant woman ask for the results of her pap smear (a test that checks for cancer of the mouth of the womb)? Choose one answer.**

1. Yes
2. No, the clinic will contact you
3. Don’t know

**7. Why do the nurses test the urine and blood pressure when a pregnant woman attend clinic for pregnancy? Choose one answer.**

1. To be sure her baby does not have a cleft palate
2. To check for a serious complication called pre-eclampsia
3. Don’t know

**8. How can a pregnant woman stay healthy during pregnancy?** **Choose as many answers as you like.**

1. Have a drink if she feels anxious about the pregnancy
2. Stop smoking
3. Eat lots of fatty food to make sure the baby grows big and strong
4. Gain at least 20 kilos to have a big healthy baby
5. Eat a healthy, balanced diet.
6. Don’t move around. Exercise is not good for the baby
7. Don’t take any drugs
8. All medicines are safe
9. Only take medicine prescribed by the nurse or midwife
10. Don’t know

**9. Why should a pregnant woman take folic acid tablets (which she gets at the clinic) during pregnancy? Choose one answer.**

1. t prevents labour too early
2. It decreases the risk of serious birth defects
3. Too much folic acid can harm the baby
4. Don’t know

**10. How do drugs and alcohol affect the baby growing in the womb? Choose as many answers as you like.**

1. They don’t affect the baby
2. During the first 12 weeks drugs and alcohol can cause major abnormalities with the baby
3. Even after the first 12 week the baby is still at risk for abnormalities
4. Don’t know

**11. Should a pregnant woman seek medical help outside her appointments at pregnancy clinic? Choose as many answers as you like.**

1. No, the sisters will check everything that needs to be checked
2. Yes, if she experiences any abnormal symptoms such as bleeding, persistent frontal headache, sudden swelling of hands, feet and face seek medical help immediately
3. Yes, if the baby does not move in 12 hours
4. Yes, if she is tired
5. Don’t know

**12. What are the signs of labour? Choose as many answers as you like.**

1. When a woman has three contractions within 10 minutes
2. When the water breaks
3. Having to go to the toilet a lot
4. Bleeding
5. Vomiting
6. Feeling the baby kicks a lot
7. Don’t know
